# Male involvement in reproductive, maternal, newborn, and child health: evaluating gaps between policy and practice in Uganda

**DOI:** 10.1186/s12978-020-00961-4

**Published:** 2020-07-27

**Authors:** Prerna Gopal, Duncan Fisher, Gloria Seruwagi, Henock B. Taddese

**Affiliations:** 1 School of Public Health, Imperial College London, Medical School Building, Norfolk Place, London, W2 1PG UK; 2 Family Included, Upper House Farm, Crickhowell, NP8 1BZ UK; 3 Makerere University School of Public Health, University Rd, Kampala, Uganda

**Keywords:** Male involvement, Reproductive, Maternal, Newborn, And child health, Uganda, Policy implementation, Resources, Skill, Community

## Abstract

**Introduction:**

Male involvement in maternal and child health is a practice wherein fathers and male community members actively participate in caring for women and supporting their family to access better health services. There is positive association between male involvement and better maternal and child health outcomes. However, the practice is not always practiced optimally, especially in low- and middle-income countries, where women may not have access to economic resources and decision-making power.

**Aim:**

This study investigates how key stakeholders within the health system in Uganda engage with the ‘male involvement’ agenda and implement related policies. We also analyzed men’s perceptions of male involvement initiatives, and how these are influenced by different political, economic, and organizational factors.

**Methodology:**

This is a qualitative study utilizing data from 17 in-depth interviews and two focus group discussions conducted in Kasese and Kampala, Uganda. Study participants included men involved in a maternal health project, their wives, and individuals and organizations working to improve male involvement; all purposively selected.

**Result:**

Through thematic analysis, four major themes were identified: ‘gaps between policy and practice’, ‘resources and skills’, ‘inadequate participation by key actors’, and ‘types of dissemination’. These themes represent the barriers to effective implementation of male involvement policies. Most health workers interviewed have not been adequately trained to provide male-friendly services or to mobilize men. Interventions are highly dependent on external aid and support, which in turn renders them unsustainable. Furthermore, community and religious leaders, and men themselves, are often left out of the design and management of male involvement interventions. Finally, communication and feedback mechanisms were found to be inadequate.

**Conclusion:**

To enable sustainable behavior change, we suggest a ‘bottom-up’ approach to male involvement that emphasizes solutions developed by or in tandem with community members, specifically, fathers and community leaders who are privy to the social norms, structures, and challenges of the community.

## Plain English summary

Male involvement in the context of maternal and child health, refers to men’s active involvement in the care of their partners and children. Where men are actively involved, research has shown improvements in health outcomes. However, the number of men who demonstrate these behaviors, such as by accompanying their pregnant spouses on antenatal care visits, is generally very low, especially in low-and-middle-income countries. Hence, global health organizations, national governments, and non-governmental organizations are advocating for increased male involvement. Through this project, we wanted to describe the barriers to male involvement in Uganda, by interviewing select individuals: men and their spouses, as well as individuals and organizations working to improve male involvement in Uganda.

We analyzed participants’ responses to identify key issues that hinder men’s involvement. Our key findings include: health workers do not always have the necessary training to support men in their partner’s health; most interventions were funded by international donors, which jeopardized lasting impact; male involvement efforts were mostly run out of clinics and not rooted within communities, and that there is a shortage of effective communication amongst key stakeholders on this topic. We encourage more homegrown initiatives drawing on cultural resources and elders in the community, to support male involvement effectively and sustainably.

## Introduction

Male involvement in the context of maternal and child health, is the practice wherein fathers and other men in the community facilitate access to better healthcare facilities and services for women and girls [26]. A man is involved if he is “*present, accessible, available, understanding, willing to learn about the pregnancy process and eager to provide emotional, physical and financial support to the woman carrying the child*” [1]. The evidence highlights the positive association between male involvement and maternal health outcomes, especially those related to the utilization of services, preparation for childbirth, and nutrition [36].

Global recognition of the importance of men in reproductive, maternal, newborn, and child health (RMNCH) can be traced back to the early nineties. The United Nations International Conference on Population and Development [43], World Conference for women [44], and the 48th UN Commission on the Status of Women [41] were responsible for bringing the male involvement agenda into focus (UN [19, 42, 45]). Consequently, there has been increasing support from the World Health Organization (WHO), United Nations Population Fund (UNFPA), national governments, and many non-governmental organizations (NGOs) towards promoting male involvement [6, 26].

The Government of Uganda has developed many policies over the last decade to directly promote male involvement in RMNCH or to encourage male involvement as part of a wider strategy related to women’s advancement. The Uganda Gender Policy of 2007, the National Policy for Elimination of Gender-Based Violence, and the National Infant and Young Child Feeding program have all highlighted the importance of the power dynamics between women and men, and that of enhancing the active participation of men through education campaigns and community involvement [19, 20, 39]. Another recent policy was the Male Action Groups or MAGs, which were initiated by the government to train and deploy men at grassroots levels to teach their peers about family planning, reproductive and maternal health services [24].

Uganda has one of the highest maternal and child mortality rates in the world. Currently, the infant mortality rate (IMR) is 45/1000 live births and maternal mortality rate (MMR) is 343/100,000 live births [40]. Around 65% of women are unable to afford treatment for complications, close to half of all women avoid antenatal care due to lack of transport, and one-fourth are hesitant to go to health facilities alone [49]. Over half of all women give birth outside of health facilities [39, 49]. Male involvement has been recognized as an integral part of the health system’s response to delays in seeking care, reaching hospitals, and accessing appropriate care [47].

Despite the clear policy emphasis, male involvement levels remain very low. A cross-sectional survey of 384 men in the Wakiso district in 2016 found that only 6% of men accompanied their wives for antenatal checks [13]. Similar observations have been reported in other regions of Uganda [38]. Studies have lamented the patchy coverage of male involvement initiatives across the country [7, 30, 35].

Globally, some qualitative studies have explored the factors that inhibit male involvement from the perspectives of men [2, 5, 7, 12–14, 18, 22, 31, 34, 37]. In these studies, the commonly identified barriers to male involvement included socio-cultural norms, gendered roles, and lack of knowledge about reproductive and maternal health. However, the literature has thus far not gauged the perspectives and experiences of key stakeholders, such as government officials, healthcare workers, and civil society. As key policymakers, the views of these stakeholders are vital to understanding implementation processes and challenges therein. Accordingly, this study seeks to address this gap by exploring the views and experiences of key stakeholders in Uganda, regarding the male involvement agenda.

## Theoretical framework

Male involvement in RMNCH is a relatively novel idea within the Ugandan health system. As such, to study the adoption and implementation of male involvement, it was imperative to focus on how the evidence has been perceived and propagated by different actors operating within the health system. We adopted a guiding conceptual framework, namely, the Promoting Action of Research Implementation in Health Services (PARIHS) framework, which was developed by the Royal College of Nursing in the United Kingdom to understand the adoption of new ideas and research into practice [29]. The framework highlights the key factors that influence the adoption of new ideas: evidence, context, and facilitation.

**Fig. 1:**
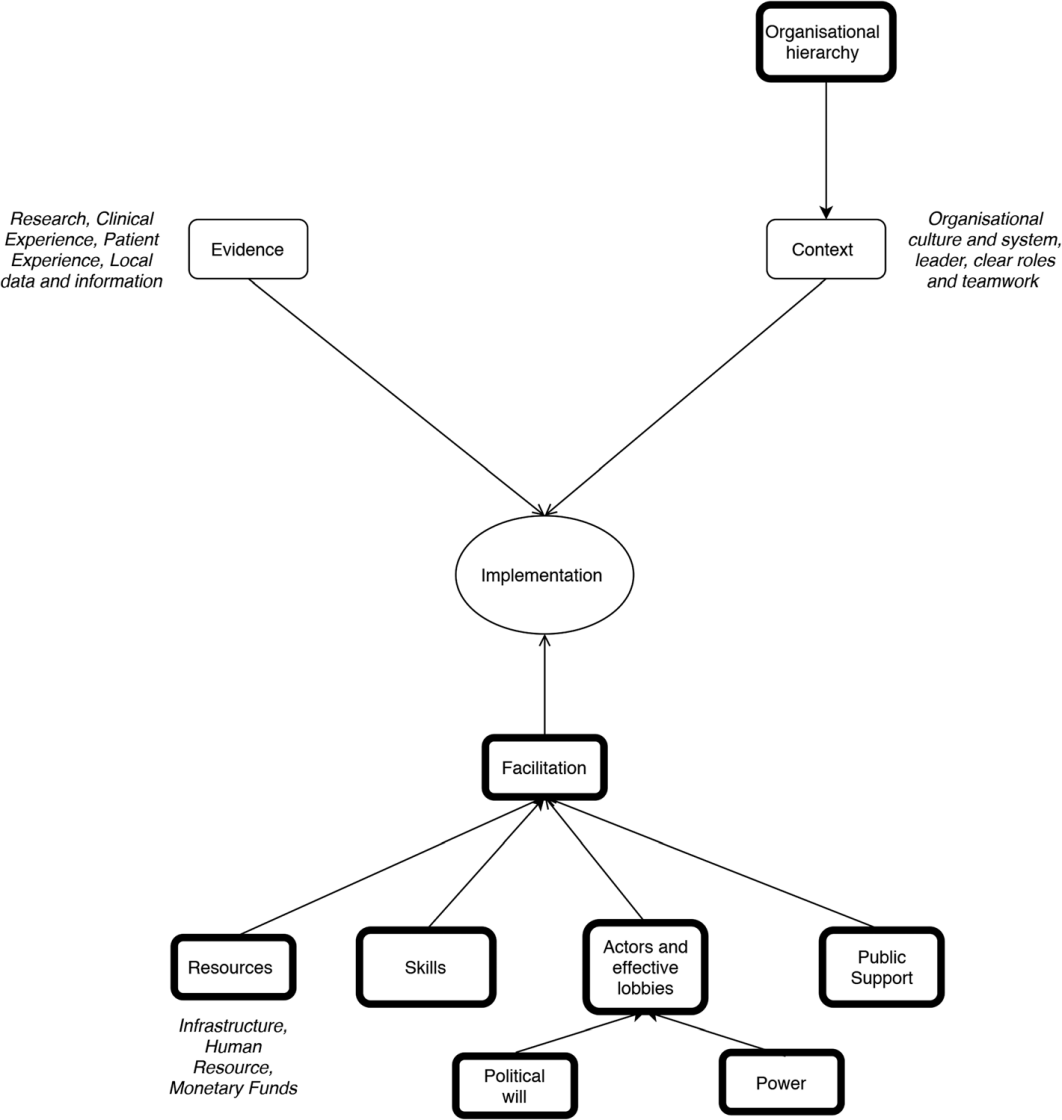
Guiding conceptual Framework adapted from the PARIHS Framework [29] and Hall et al’s theory on Agenda Setting [9]

Furthermore, according to Hall et al., a policy agenda is more likely to be successful if it includes three factors: legitimacy, feasibility, and support [9]. Although there is an overlap between the legitimacy (Hall) and evidence (PARIHS), the PARIHS framework does not include feasibility or support which are crucial factors in the implementation process. Hence, these were included in our adaptation of the PARIHS framework along with the power distribution and political will of actors, which have been identified by key health systems and policy literature as critical factors [28]. Therefore, our guiding theoretical framework incorporates core concepts from the PARIHS Framework (context, evidence, and facilitation), alongside feasibility (resources and skills), public support, and the power relationship between actors (Fig. 1).

## Methods

We employed a case study approach, following Yin’s definition of a ‘case study as “an empirical inquiry that investigates a contemporary phenomenon in depth and within its ‘real-life’ context especially when the boundaries between phenomenon and context are not clearly evident” [50]. We conducted a case study of a male involvement project, namely, the Emanzi project, which was designed and implemented by Family Health International 360 (FHI360) as one of the few male involvement programs in Uganda.

The Emanzi intervention evolved from another pre-existing, multi-country intervention called ‘Men as Partners’, developed and implemented by Engender Health International. The Emanzi project (‘emanzi’ means ‘champions’ in the local language) was then modified for the Ugandan context and implemented in three south-western Uganda districts - Kasese, Kamwenge, and Kanungu, in 2014. The project mainly involved working in tandem with the local governmental authorities to conduct learning-and-knowledge sessions for fathers who had or were about to have children. These sessions were conducted over 9 months and involved a wide range of topics from family planning to HIV.

We followed a purposive, maximum variation sampling method to ensure the inclusion of different types of stakeholders and viewpoints [32]. We conducted 17 interviews with policymakers, academics, healthcare workers, and representatives from multilateral organizations, and two focus group discussions (FGD) with participants of the Emanzi project and their spouses. We recruited policymakers (academics, multilateral organizations, and NGO representatives) through email invitations. The fathers and women groups were recruited through announcements by, and referrals from the Emanzi project. Religious leaders and healthcare workers in Kasese and Kampala were identified via snowballing during interviews and through contacts established in and around the Emanzi project.

The breakdown of the type of participants is provided in Table 1 below.

**Table 1:** Breakdown of participants by position and organisation

Participant number	Position	Organisation
1	Healthcare Worker and Project Implementor	NGO
2	Project Analyst	NGO
3	Representative Secretary	Multilateral Organisation 1
4	Academic Researcher	Government University
5	Project Analyst	NGO
6	Programme Director	NGO
7	National Programme Officer	Multilateral Organisation 2
8	Programme Coordinator	Multilateral Organisation 2
9	District Health Officer	Government of Uganda
10	Rural Health Worker	Government of Uganda
11	Church Leader	Local Church
12	Rural Nurse and Family Planning coordinator	Government of Uganda
13	District Health Coordinator	Government of Uganda
14	Senior Nurse	Government Hospital
15	Senior Consultant	Ministry of Health
16	Rural Healthcare worker and medical doctor	Government of Uganda
17	Rural Healthcare worker and medical doctor	Government of Uganda
FGD 1	Male participants of the Emanzi Intervention	Residents of Kasese
FGD 2	Wives of participants of the Emanzi Intervention	Residents of Kasese

Kasese was more accessible to the project team given the limited time for fieldwork. Multiple category design, which involves multiple homogenous groups of participants, was adopted to allow data collection and comparison across two groups, one with fathers and the other with their spouses. This approach allowed for rapid data collection and was well suited to understand deeply contextual topics [15].

Each interview took up to 1 hour and was conducted at the participant’s office, while the FGDs were conducted in a community center in Kasese. The interviews with national-level policymakers were all conducted in English. For the FGDs with the fathers and their spouses, we used translators, as well as translating copies of key documents such as the project information sheets and consent forms. The translations were checked by a member of the research team who is bilingual. All participants were fully informed about the objectives and expected outcomes of the research project. Ethical clearance was obtained from The AIDS Support Organization Research Ethics Committee in Uganda and the Research Ethics Committee at Imperial College London.

All interviews were promptly transcribed to allow continuous review of emerging themes and subsequent interviews. This was followed by a thematic analysis that drew upon the apriori framework as well as incorporating new emergent themes from the data. The data were manually coded by the primary author. Primary themes were identified and grouped into broader themes based on their likeness or conceptual homogeneity [17]. This process was repeated until 4–5 major themes emerged.

### Researchers’ positionality

This study draws on the feminist philosophical perspective. The questions asked, and the subsequent analysis were guided by our strong stance regarding the enhancement of male involvement as a means for more egalitarian conditions around maternal health and newborn and childcare.

All four authors come from varied backgrounds and countries – yet they strongly disapprove of hegemonic masculinity and its repercussions on society. The primary author comes from a deeply patriarchal society that subjects women to many forms of oppression and discrimination. However, the author’s liberal upbringing within her family and egalitarian values fostered a strong belief that men want to be active partners and involved-parents. Beyond this, we uphold a ‘non-deficit’ perspective, which situates the challenges of male involvement beyond men, within wider social structures and health systems [10]. We are aligned with the notion that men’s participation is limited by social and economic constraints and archaic gender norms [21].

## Findings

Four major themes emerged from the data: ‘gaps between policy and practice’, ‘resources and skills’, ‘inadequate participation by key actors’, and ‘types of dissemination’. The first theme, ‘gaps between policy and practice’, was the only theme that did not correspond to concepts specified apriori within the conceptual framework described in Fig. 1.

### Theme 1: gaps between policy and practice

On the whole, there was a shared understanding amongst participants regarding the multifaceted nature of the male involvement agenda. They described it as a holistic involvement and active support rendered by men towards maternal and childcare needs. A response from an NGO participant exemplifies this:

*It is beyond just presence like escorting the wife to the hospital … it has both the element of the man being able to communicate more effectively with their spouse and being supportive in their home [Participant 6, NGO].*

However, the current policies and programs are seen to be reinforcing the singular message of fathers and husbands accompanying their spouses to health facilities. This is seen as the practical step that is permitted in the context, whereas engaging men and communities with the broader messaging is seen to be complex. A participant went on to explain this by saying:

*The men are not easy to get by [to]. They are always saying that they are busy looking for money... But, as a compromise if they are at least accompanying them for at least antenatal [care visits], if they come for at least one visit and they are counseled and tested for HIV/AIDS then it would be okay [Participant 15, Government].*

Participants went on to highlight that this practice, which limits the conception of male participation to just accompaniment, instigates a less than optimal and unanticipated behavioral response. As health centers incentivize antenatal care attendance by prioritizing couples over single mothers, some mothers are said to appear at health centres with random men they might have just met, commonly motorbike taxi drivers, just to beat the queues.

*We had issues of women hiring boda-boda [motor bike taxis] men because it was like “If I don’t go with my man then I will be in trouble with the midwife. If I go with a man and say that he is my husband I will be looked at faster” [Participant 8, Multilateral Organisation 2].*

Participants felt that this trend fell short of actual participation or behavior change. They further discussed how the impact of this approach is bound to be short-lived.

### Theme 2: resources and skills

Participants highlighted the shortage of male-friendly reproductive and sexual health services in Uganda. They explained that RMNCH is still widely viewed as a ‘women’s issue’. The physical space of maternity wards was described as a ‘dormitory’ where there was no privacy to encourage men to attend and meaningfully participate in the care of their partner. Nurses were said to lack an inclusive stance towards men. Interviewed nurses mentioned that they did not receive any training or guidelines on how to integrate men into their services and how to make the facility male friendly. Five health workers reported that there are no national guidelines for mobilization of men. The existing guidelines were limited to providing basic information about contraception, reproductive health, and sexually transmitted diseases.

*Neither were health workers trained to receive men nor do they do anything for men when they come. Men weren’t allowed inside, and it wasn’t convenient. Even now they really struggle with what to do with men? Because a lot of the health facilities are like dormitories and even maternity wards are quite like dormitories so then how do you bring in a man? [Participant 4, Academic].*

Most participants, particularly the health workers, talked about the lack of adequate resources, infrastructure, and space. In these circumstances, integrating men into maternity and child-care services becomes a challenge. Furthermore, short-staffed and overworked health workers find it difficult to incorporate strategies for encouraging male participation. The following quotes vividly portray the impact and importance of resources concerning male involvement.

*We are congested. There is no way you can allow your husband to be in the labor ward because if you went in, so many women pushing in. You can’t allow. If we improve our labor wards so that at least each mother delivers from her own room and there is privacy then the husbands could be allowed [Participant 15, Ministry of Health].*

*We are talking about, recently we reported 1 doctor per 16,000 people, 1 nurse to 6000 people. So, when you look at the ratios, the capacity is not there. Unless you are telling me that they are going to recruit more staff [Participant 16, Rural health worker].*

Another key challenge discussed by more than half the participants relates to the heavy dependence of the government on external aid and support. Many rural healthcare workers raised concerns about the sustainability of donor-funded projects. Male involvement was often seen as a donor-driven agenda and the uncertainties of funding inhibit systematic and sustained implementation. Most male involvement projects were implemented in regions that overlap with the interests of donor organizations thereby leading to patchy implementation across the country.

*It (program) has to be sustainable because now if they pull out; men will go back. We need to look for interventions that can be sustained for a long time. Even for generations [Participant 17, Rural health worker].*

*Our health service, you realize that about a third or 40%, that is an estimate, is dependent on them (donor funds). But I don’t think they [the Government] have thought about this because they don’t have the capacity [Participant 16, Rural health worker].*

### Theme 3: inadequate participation by key actors

Most participants including NGO officials and rural health workers indicated that the government showed political will to accommodate men in RMNCH. The Ugandan government has also been working with other stakeholders to bring about change, especially international organizations and NGOs.

*The will at the government level is there. They speak to it. The President and his wife, the ministries speak to it. The will is there but the action will take time [Participant 7, National Programme Officer].*

*Government has come in with different stakeholders to say it’s not just a woman’s issues, it’s a partnership, let’s work together for the betterment and health of both of us (organizations and populations) [Participant 8, Multilateral Organisation 2].*

However, most of these collaborations and programs are suspended at the level of international organizations and the Ministry of Health. Even though these are powerful and influential players in the health sector, their interaction with the community at the grassroots level is often limited. In contrast, religious leaders, political leaders, and local health teams would be better positioned to advance this culturally embedded behavior. Participants especially emphasized the importance of religious leaders as conduits of social change. This was especially highlighted as crucial to ensure the sustainability of the behavior change within the population.

*If you want to sensitize the public that’s how you need to involve various sectors. Can we bring the religious leaders on board? Because they understand very many problems. Can we bring those leaders and inform them of the importance of having these men participate? [Participant 17, Rural health worker].*

### Theme 4: manner of dissemination (Organisational hierarchy)

We observed a gap in the conception and propagation of ideas between policymakers (chiefly, the Ministry of Health and donors) and agents of delivery, such as health workers including doctors, rural health workers, village health teams and nurses. Participants felt that directions were often handed down the chain, rather than a sense of empowerment engendered through continuous engagement and capacity building. Furthermore, the propagation of these ideas and directives were not supported by a structured and detailed message in terms of how the idea should be implemented. Specifically, participants highlighted the absence of guidelines and related training to enable male participation.

*The government has actually tried this but the fact that the government doesn’t empower people at the grassroot to reach men means it is failing but it can emulate what Family Health International 360 has done by empowering village health teams (VHTs) to train men within reach. Then the government can also possibly succeed if they can train VHTs to train men within easy reach [Participant 13, District Health Coordinator].*

In addition, suboptimal dissemination was evident as some key policy actors were unaware of the Emanzi project. As a flagship initiative on male involvement, one would expect widespread awareness; lack of which signifies ineffective dissemination.

## Discussion

In this study, we explored how various actors engage with the male involvement agenda and the factors that influence its implementation in Uganda. In doing so, we developed four major themes: ‘gaps between policy conception and practice’, ‘resources and skill’, ‘inadequate participation by key actors’, and ‘manner of dissemination of idea’. While the importance of male involvement was accepted by all participants, the translation into action was inconsistent. The themes also emphasize the importance of organizational hierarchy and the influence of actors on agenda setting and policy implementation. The agenda is disseminated in a top-down fashion, from government and donors down to local actors. In a context where informal care and traditional healing plays a major role, strategies for behavior change should involve religious leaders and elders. Across these themes, the overarching message relates to the need for a comprehensive and bottom-up approach towards male involvement, that is, solutions that are developed by or in collaboration with community members, particularly, fathers and community leaders who are privy to the social norms, structures, and challenges of the community.

For any policy to be successfully implemented, the dynamics between the various organizational hierarchies and their capacity is critically important [8]. The relationship and communication between policymakers on the one hand, and implementors, on the other, holds the key to achieving intended outcomes. Conversely, the lack of clarity of concept and sub-optimal engagement and feedback between these actors impairs the implementation of policies and results in the omission of influential actors such as traditional and religious leaders. Also, drawing on community resources would further reduce the observed dependence on external aid and improve the sustainability of health behavior [3, 11].

Similar gaps between policymakers and implementers have been well documented within other health systems. A qualitative study conducted in Lesotho by Vian & Bicknell deployed the Principal-Agent Theory to evaluate a program on performance-based budgeting and explored the different sets of actors specified in the theory: the principal, intermediary officials, and the agents [4, 46]. Similar to our study, they identified a lack of training, the absence of guidelines, and inadequate capacity as major problems leading to weak implementation of policies and programs; all of which are pertinent to the male involvement intervention explored in this study [46].

The finding that community engagement is essential to encourage male participation replicates the results of multiple studies that have been conducted globally, highlighting the positive influence of community-based interventions, particularly those involving community health workers (CHWs) [16, 25, 33, 48]. However, the mere participation of religious leaders, political leaders, and CHWs is not enough [27]. It must be met with an improved capacity of facilities and training of health professionals to achieve positive results. Echoing our observations, Kaye et al. conclude that men find it difficult to participate in maternal and child health issues due to the congestion and poor state of health facilities [14].

Finally, we found it encouraging to see the positive views of actors towards male involvement. Our findings contradict the observation of Sileo et al. [31] that there is low interest amongst key stakeholders towards male involvement. It may well be that participants in our study have more favorable views for having been sensitized through the EMANZI project. Still, there is a need to sustain the positive perceptions and deepen the engagement beyond mere tokenistic gestures into more holistic involvement of men in the health of women and newborns. There is also need to develop resources and skills, standardize practice and share ideas, and engage the community and cultural leaders for a system-level approach to enable change. In practice, health systems strengthening efforts need to streamline key concepts such as male involvement as they work towards developing the building blocks of the health system such as human resources, infrastructure and financing, health services, health management information systems, technology, and governance. In terms of health care financing, the reliance on external aid would need to be minimized to ensure more sustainable ways of driving the agenda.

We employed a multi-stakeholder analysis, using interviews and focus group discussions, which enabled us to identify multiple perspectives. We undertook a theoretically grounded thematic analysis, which enhances the transferability of the findings to similar settings. The use of a translator during the focus group discussions is a limitation of the study as the translation-based interviews may generate less robust findings. As a consequence, the outputs of the focus groups were limited and could only be put to limited use within this study; mainly to triangulate the findings of in-depth interviews with stakeholders. Some potential participants could not be contacted due to the requirements of extra administrative approvals; we overcame this limitation by interviewing other individuals from similar organizations or health facilities. Finally, interviews with influential members of the community and health system need to be interpreted with caution as they are likely to involve micro-political agenda on the part of policymakers [23]; we deployed a method of triangulation along with probing questions to ensure that we have captured contrasting views.

## Conclusion

Through this study, we highlight the need for more sustainable male involvement interventions that improve the dissemination of the concept more effectively and standardize implementation across different levels of actors. Specifically, the key policy actors, namely the government and donors, need to establish buy-in from and effectively engage community members, and religious leaders in the male involvement agenda. Furthermore, health workers need to be supported in a structured manner, including through training and guidance, to implement the idea optimally. We observed a general sense of enthusiasm for the male involvement agenda. What remains is a structured and bottom-up approach to ensure optimal practice.

## Data Availability

Not applicable.

## Abbreviations

CHWs: Community health workers
CPIPO: Commission on Paternal Involvement in Pregnancy Outcomes
FGD: Focus group discussions
FHI360: Family Health International 360
IMR: Infant mortality rate
MMR: Maternal mortality rate
MOGLSD: Ministry of Gender, Labour and Social Development
MTCT: Mother-to-child transmission of HIV
NGOs: Non-Governmental Organisations
PARIHS: Promoting Action of Research Implementation in Health Services
PAT: Principal-Agent Theory
RMNCH: Reproductive, Maternal, Newborn, and Child Health
TBA: Trained Birth Attendants
UBOS: Uganda Bureau of Statistics
UNFPA: United National Population Fund
VHTs: Village Health teams
